# Mosquito Population Regulation and Larval Source Management in Heterogeneous Environments

**DOI:** 10.1371/journal.pone.0071247

**Published:** 2013-08-07

**Authors:** David L. Smith, T. Alex Perkins, Lucy S. Tusting, Thomas W. Scott, Steven W. Lindsay

**Affiliations:** 1 Department of Epidemiology, Johns Hopkins Bloomberg School of Public Health, Baltimore, Maryland, United States of America; 2 Malaria Research Institute, Johns Hopkins Bloomberg School of Public Health, Baltimore, Maryland, United States of America; 3 Fogarty International Center, NIH, Bethesda, Maryland, United States of America; 4 Department of Entomology, University of California, Davis, California, United States of America; 5 Department of Disease Control, London School of Hygiene and Tropical Medicine, London, United Kingdom; 6 School of Biological and Biomedical Sciences, Durham University, Durham, United Kingdom; University of Texas Medical Branch, United States of America

## Abstract

An important question for mosquito population dynamics, mosquito-borne pathogen transmission and vector control is how mosquito populations are regulated. Here we develop simple models with heterogeneity in egg laying patterns and in the responses of larval populations to crowding in aquatic habitats. We use the models to evaluate how such heterogeneity affects mosquito population regulation and the effects of larval source management (LSM). We revisit the notion of a carrying capacity and show how heterogeneity changes our understanding of density dependence and the outcome of LSM. Crowding in and productivity of aquatic habitats is highly uneven unless egg-laying distributions are fine-tuned to match the distribution of habitats’ carrying capacities. LSM reduces mosquito population density linearly with coverage if adult mosquitoes avoid laying eggs in treated habitats, but quadratically if eggs are laid in treated habitats and the effort is therefore wasted (i.e., treating 50% of habitat reduces mosquito density by approximately 75%). Unsurprisingly, targeting (i.e. treating a subset of the most productive pools) gives much larger reductions for similar coverage, but with poor targeting, increasing coverage could increase adult mosquito population densities if eggs are laid in higher capacity habitats. Our analysis suggests that, in some contexts, LSM models that accounts for heterogeneity in production of adult mosquitoes provide theoretical support for pursuing mosquito-borne disease prevention through strategic and repeated application of modern larvicides.

## Introduction

Dynamic models of malaria transmission have influenced strategic decisions about disease prevention from the time of Ronald Ross in the early 20^th^ century, when larval source management (LSM) was the dominant form of vector control [1]. After early field deployment of DDT demonstrated that indoor residual spraying (IRS) was an extremely effective way to control malaria, George Macdonald’s influential mathematical analysis showed that transmission was highly sensitive to adult mosquito mortality rates [2]. This analysis and emerging theory reinforced the prevailing notion at the time that DDT was a sufficient tool for malaria eradication [3], [4], and IRS was implemented largely to the exclusion of LSM. The legacy of Macdonald’s sixty-year old analysis can be seen in contemporary policy decisions by leading international organizations, including a recent evaluation by the World Health Organization (WHO) that was highly critical of larviciding in sub-Saharan Africa [5]. These recommendations, based largely on the Ross-Macdonald model that lacks dynamic mosquito populations and is ill suited to evaluate LSM, come despite evidence that LSM can achieve similar results and at a similar cost to ITNs [6]. Here, we re-examine the simple models that have motivated such analyses, and we derive some basic lessons for mosquito population dynamic and control to guide policy for LSM.

Several mosquito population dynamic models have been developed that link adult and immature aquatic populations [7]–[11], and a few have explicitly considered LSM [12]. Most models of larval populations, whether simple or complex, make some assumption about density dependence and population regulation. Some have considered the complex structure that arises from having populations of eggs, four larval instars, and pupae [13]. Others have considered the dynamics of systems with predators or resource-based competition [14]. Complicated computer-simulation models have considered the effects of heterogeneity in rainfall and temperature, heterogeneous habitat geometries with variable responses to flushing, and desiccation [9], [10], [14]–[17]. Finally, a few models have considered how the distribution of larval habitat constrains egg laying and affects the adult mosquito population dynamics and pathogen transmission [18]–[20]. It remains unclear how heterogeneity affects the way mosquito populations are regulated and what variation in key processes means for LSM. Here, we present a simple theoretical framework that can be used to understand habitat heterogeneity, the local effects of density dependence, the factors that affect the outcome of LSM in dynamic, heterogeneous environments, and their total effects on pathogen transmission.

## Methods

Many factors have been implicated in immature mosquito population dynamics, including egg laying, water temperature, resource limitations, predation, larval development rates, the ephemeral availability of mosquito habitat due to evaporation and desiccation, or filling or flushing habitat from the combination of rainfall and hydrology [21]. Here, we take a simpler approach that focuses narrowly population regulation when aquatic habitats are heterogeneous. The model may not be suitable for some purposes, such as simulating mosquito population dynamics when realistic lags for mosquito development are required (i.e. see [13]), but the models do provide insights into the regulation of mosquito populations, population dynamics in heterogeneous habitats and the effects of LSM. These lessons can, perhaps, serve as a theoretical basis for understanding more complicated models.

### The Mosquito Population Dynamic Model

The following model considers the coupled dynamics of aquatic immature and terrestrial adult mosquito populations. We assume the population of larval mosquitoes is subdivided into 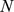 distinct aquatic habitats. Individual aquatic habitats are hereafter called “pools” to facilitate communication, even though this may not be the best description of many kinds of larval habitats.

Let 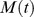 denote the population density of adult mosquitoes at time 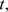 and let 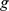 denote the per-capita death rate. The number of larvae in each pool at any given time is denoted 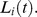 Let 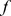 denote the mosquito blood feeding rate, 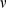 the number of eggs laid by a mosquito each egg laying cycle, and 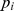 the fraction of eggs laid in the 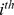 pool. In the 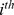 pool, mosquitoes are assumed to mature at rate 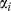 and die at the per-capita rate 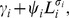 where 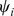 represents a pool-specific increase in per-capita mortality in response to crowding. For 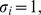 which was assumed for most of our analysis, the relationship gives mean crowding, which is analogous to the classical first-order description of density dependence as described by the logistic growth equation. Under these assumptions, mosquito population dynamics are described by the following equations:
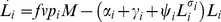
(1)

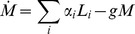
(2)


Homogeneous environments were defined by letting each pool have identical parameters and by distributing eggs evenly among the pools. Environments were made heterogeneous by varying parameters describing larval dynamics or egg laying (i.e. 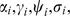 or 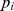 from Eq. 1) strategically to illustrate specific aspects of this system. We constructed completely heterogeneous environments by drawing random numbers for all larval dynamic parameters and for egg laying. Parameter names are summarized in Table S1 along with all the values used for the simulations. The population dynamics in these completely heterogeneous environments depends on some notion of the response to crowding, the distribution of eggs laid, and the potential capacity for adult mosquito production of each pool.

### Larval Source Management

LSM was simulated by assuming that control was applied either permanently or repeatedly to a subset of these pools, which were called “treated.” This is done to make the analysis simpler and to illustrate properties of the models, even though there might be real constraints on the ability to completely and permanently nullify mosquito productivity. Coverage was defined as the proportion of aquatic habitats that were treated. In our simulations, LSM was assumed to prevent all larval development and eliminate productivity such that no adults emerged from treated pools. The analysis focused on the relationship between coverage and the “control effect size” on transmission, defined for LSM as the proportional decline in the adult mosquito densities when compared to the same system without control.

The control effect sizes of LSM were simulated under two different assumptions about changes in egg-laying behaviour of adult mosquitoes in response to LSM. First, mosquitoes could continue to lay eggs in the pools that had been treated, such as when modern non-repellent larvicides are applied to aquatic habitats. Second, mosquitoes could avoid treated pools and lay eggs elsewhere, either because larvicides in the water acted as repellents or because the habitat was modified or destroyed.

Control effect sizes of increased LSM coverage were examined for five classes of population dynamic simulations based on different assumptions about crowding and egg laying: (1) a homogeneous environment where all pools have identical parameters and eggs are laid evenly; (2) a simple extension of the homogeneous model in which a fraction of habitats in a homogeneous environment were simply non-productive, so that the fraction of eggs laid in productive habitats summed to less than 1; (3) LSM was applied in random order in a completely heterogeneous environment; (4) LSM was “targeted” by treating subsets of the most productive pools in a completely heterogeneous environment (this was done in a perfectly efficient order, such that as coverage increased, the pools with highest productivity were treated first); and (5) to show a contrast, LSM was then inefficiently targeted in a completely heterogeneous environment by treating subsets of the least productive pools.

## Results

### Mosquito Population Dynamics

The key dynamic feature of the equations describing mosquito population dynamics is that emerging adult mosquitoes become part of an adult mosquito population and that they distribute eggs among many independent pools (Eq. 2). Because of many factors affecting the distribution of eggs in habitats of differing qualities, including the patterns of blood feeding, different patterns emerge from examining the dynamics of completely heterogeneous systems compared with homogeneous systems.

The “carrying capacity” was defined as the equilibrium density of larvae in a system with only one pool or in a homogeneous system, and it is given by the formula:
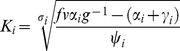



In the homogeneous system, carrying capacity determines the maximum productivity of each pool, the emergence rate of adult mosquitoes 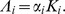


**Figure 1:**
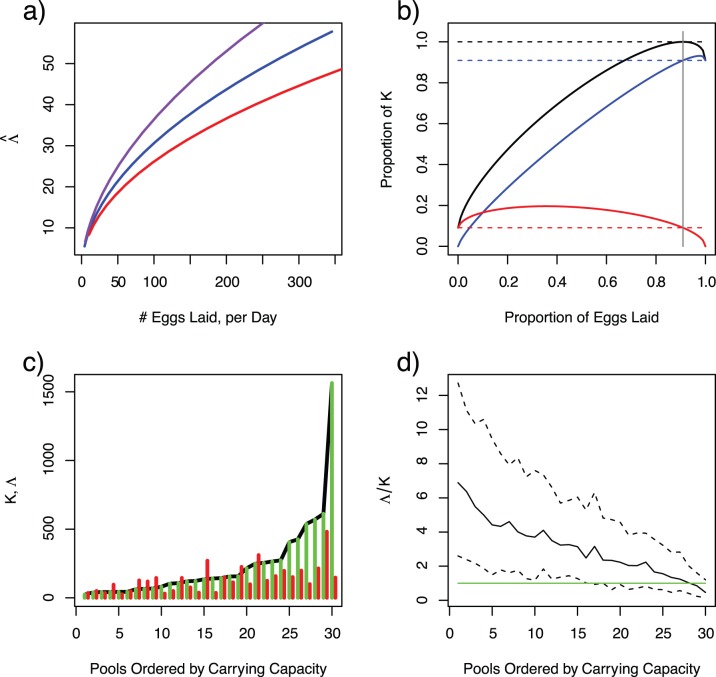
Understanding productivity (*i.e.*, the emergence rate of adults 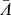) in heterogeneous habitats depends upon understanding the relationship between egg laying, carrying capacity (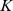), and crowding. **a**) The functional relationship between the rate of egg-laying and productivity depends on the functional response to crowding. In this model, the relationship is sensitive to the power-law scaling relationship (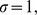 blue; 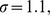 red; 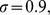 purple). Carrying capacity is given for a single value of egg laying rates, given at the steady state if that pool had existed in isolation. **b**) In a system with 2 pools linked by egg-laying, where the carrying capacity of pool 1 is approximately 90% of the total (dashed blue line) and pool 2 has the rest (dashed red line), the population totals overall (solid black) are generally below the maximum, unless egg laying is fine-tuned such that the proportion of eggs laid was equal to that pool’s proportion of carrying capacity (vertical grey). **c**) A comparison of productivity (red) and carrying capacity (black line) for a typical set of heterogeneous aquatic habitats. Productivity equals carrying capacity when the distribution of eggs laid is finely tuned to match the distribution of carrying capacities (i.e. 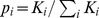). **d**) The ratio of productivity to carrying capacity was computed for 100 sets of heterogeneous aquatic habitat. The green line plots the 1∶1 ratio, when productivity equals carrying capacity. These distributions, plotted here as the median (solid line) and the 10^th^ and 90^th^ quantiles (dashed lines), shows the robust pattern that the habitats with the lowest productivity tend to be under capacity and the few highly productive habitats tend to be over capacity.

When egg-laying patterns are heterogeneous, larval density becomes decoupled from carrying capacity. The number of eggs laid and the mean crowding of each habitat affect larval densities. At the steady state, the number of eggs laid in a pool is 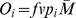 and:
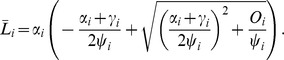



The number of adults emerging from any individual pool (*i.e.*, productivity), 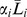 depends on the number of eggs laid and the functional relationship that determines how larval mosquito mortality increases with crowding (Fig. 1a). (Different rules would likely follow from considering predation or other types of regulation that respond dynamically to larval population density.) These productivity curves show that carrying capacity is but one point along a continuum of adult output rates from a pool in relation to the egg input rate. Larval populations thin in response to crowding, so that the proportion of adults emerging from individual pools decreases with the number of eggs laid, but the number of adults emerging increases. Dynamics of individual pools linked by egg-laying females do not, therefore, generally conform to the rules of logistic growth. In heterogeneous environments, productivity and carrying capacity are therefore not generally given by the same quantity.

Some properties of the general system come from exploring a simple system of two pools with different carrying capacities. By varying the proportion of eggs laid in each pool, productivities of the individual pools and of the whole system were compared. In this system, the total productivity equals the total carrying capacity only when the proportion of eggs laid in each pool is equal to that pool’s carrying capacity as a fraction of the total of all pool’s capacities (*i.e.*, if 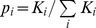, Fig. 1b).

Numerical simulations demonstrate that this rule holds in completely heterogeneous systems (Fig. 1c). In that case, total productivity is equal to the total carrying capacity only when the distribution of egg laying is fine-tuned to equal the relative distribution of carrying capacity. Unless the proportion of eggs laid is fine-tuned to match the carrying capacities, larval densities will be different than carrying capacity (Fig. 1c,d), often by a large margin. The net effect of this mismatch is unpredictable, but it will depend strongly upon the proportion of eggs laid in the most productive habitats. For the mathematical assumptions made in this model, productivity was lower than capacity in approximately one-third of the cases, but productivity often exceeded carrying capacity. In at least one case, productivity exceeded capacity by 270%.

Intuitively, the dynamic interplay of mobile adults, distributed aquatic habitats, and the response to crowding means that total productivity is strongly affected by the correlation between the distribution of eggs laid and the distribution of carrying capacities in aquatic habitats. The proportion of eggs that survive to become adults in any one pool is reduced as egg laying increases crowding, but the number of eggs being laid depends on the whole ensemble of aquatic habitats. Pools that receive the most eggs will tend to have population densities that exceed their carrying capacities, while those that have the fewest eggs are usually below capacity (Fig. 1d). Crowding will be uneven and the effects of crowding in just a few pools dominate population regulation.

**Figure 2:**
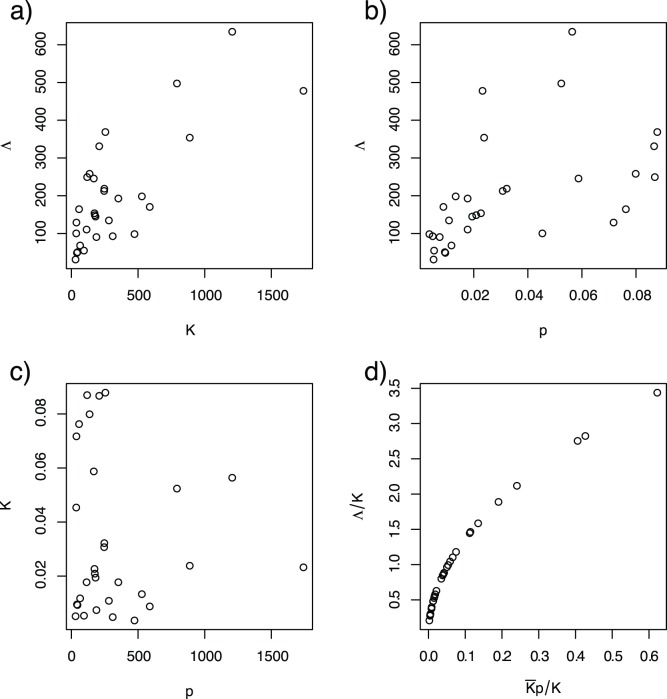
The scaling between egg-laying and productivity is only apparent after normalizing both productivity and egg laying by carrying capacity. In completely heterogeneous environments, there may be a poor correlation between **a**) carrying capacity and productivity; **b**) egg laying and productivity; and **c**) egg laying and carrying capacity. **d**) The crowding law governing density dependence is found by plotting the ratio of eggs laid to carrying capacity against the ratio of productivity to carrying capacity (i.e. 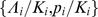). The constant 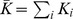 was used to scale the *x*-axis.

Productivity and carrying capacity should both be correlated with egg-laying (Fig. 2a–c), but the underlying functional relationship between eggs in and adults out is only revealed by plotting the ratio of egg-laying to carrying capacity against the ratio of productivity to carrying capacity (Fig. 2d). Though enlightening, this relationship may not have any practical use unless it is possible to measure carrying capacity directly, perhaps through surrogate measures such as a pool’s surface area, volume, or key resource levels through bioassays [22]. The notion of a carrying capacity is, therefore, useful both conceptually and theoretically. Capacity is not, however, what is typically observed in individual pools or in populations at the steady state. Instead, productivity is determined by the carrying capacities of the individual pools, heterogeneity in egg laying proportions, and the mismatch between the two patterns.

Another important principle is that, in the absence of immigration from pools outside the study area, the dynamic feedback between egg-laying and aquatic population dynamics is subject to a threshold phenomenon governing mosquito persistence. A sufficient condition for mosquito persistence is 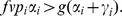 The mosquito population can, in theory, persist if at least one adult male and female mosquito is expected to emerge from a pool from an egg laid by a typical single adult mosquito originating from that pool (Analysis S1).

### Larval Source Management

The control effect sizes of LSM depend strongly upon the adult female mosquito’s egg-laying behaviour in response to LSM. The most important difference is whether mosquitoes continue to lay eggs in treated habitat. If mosquitoes avoid laying eggs in the treated habitats, then LSM simply reduces the amount of habitat available. The outcomes tend to be consistent with a common use of Macdonald’s formula for *R_0_* with respect to LSM, which assumes linear responses. The dynamics of LSM with heterogeneous biting and targeting are more complicated, however, and different rules govern systems in which adults continue to lay eggs in treated pools. Some general aspects of LSM are best illustrated in homogeneous systems, but other aspects play out differently in heterogeneous systems.

**Figure 3:**
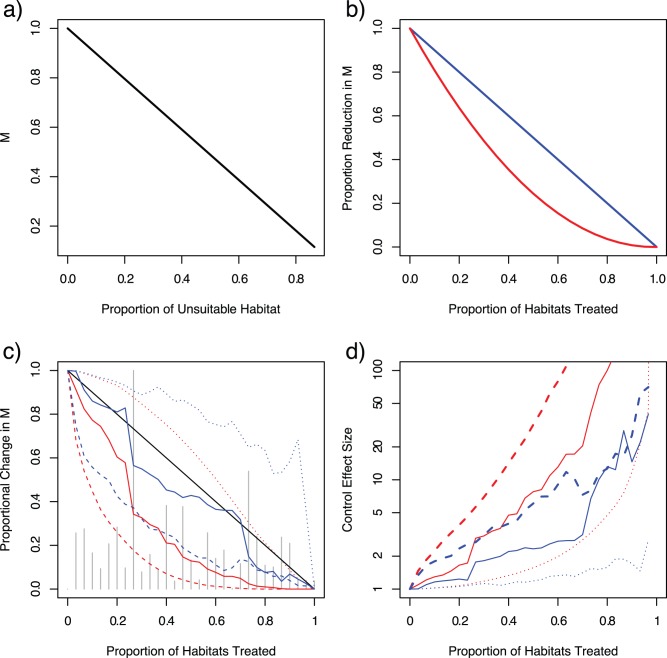
The “effect size” of LSM in relation to coverage tend to be either linear or quadratic depending on whether eggs are laid in “treated” habitats and how well LSM is targeted. **a**) Holding the total number of productive pools constant, adult mosquito population density declines as the number of unproductive pools increases and absorb eggs. **b**) The “egg sink” effect gives a non-linear effect to LSM if adult mosquitoes continue to lay eggs in the treated pools, so that treating 50% of the pools reduces adult density by 75%, and treating 75% of the pools reduces adult density by 95% (red). If adult mosquitoes do not lay eggs in the treated pools, however, then reductions in mosquito density are proportional to the % of habitat treated (blue). **c**) The change in adult mosquito density due to LSM in highly heterogeneous habitat as a function of the proportion of habitats treated depending on whether the adults lay eggs in treated pools (red) or avoid treated pools (blue), and depending on whether LSM was done in one particular random order (grey spikes), perfectly efficiently targeted (dashed lines), or perfectly inefficiently targeted (dotted lines). The black line represents a linear response with respect to coverage. **d**) For the same graphs as 3c, the effect sizes are plotted on a semi-log scale to highlight the benefits of LSM at high coverage. The best case for this system, with efficient targeting and egg-sink effects, predicts a hundred-fold (99%) reduction in mosquito density for 60% coverage. These benefits also get larger for higher coverage and show that there is enormous potential for LSM to reduce transmission through targeted repeated application of modern larvicides.

LSM is more effective when mosquitoes continue to lay eggs in treated habitats. To illustrate why this behaviour changes the control dynamic, consider a simple heterogeneous system in which pools either have all the same carrying capacity, or they produce no adults at all. Holding the number of productive pools fixed, the total productivity of the system declines linearly with the number of unproductive habitats. The existence of unproductive aquatic habitats nearby can thus become “egg sinks” [23], [24] and reduce the proportion of eggs laid in the productive pools (Fig. 3a).

To illustrate the relationship between coverage and control effect sizes, LSM was simulated in a homogeneous system with varying coverage levels and with both types of egg-laying responses. In these simulations, when adult mosquitoes continue to lay eggs in treated habitats, there are two effects of LSM. One effect, the reductions in the amount of productive habitat, is complemented by a second effect, an increase in the amount of habitat that serves as a sink for eggs. The two effects are multiplicative, so control effect sizes scale with LSM coverage in a way that is approximately quadratic: removing 50% of habitat reduces mosquito densities by approximately 75%, and removing 80% of the habitat reduces mosquito densities by approximately 96% (Figure 3b).

Similar results occur when habitat is heterogeneous, but the interpretation of “coverage” must be considered in a more nuanced way. In homogeneous systems, coverage describes reductions in capacity, productivity, and egg laying. In heterogeneous systems, however, the mismatch between productivity, capacity, and egg laying means that varying amounts of these three quantities remain as coverage increases. The control effect sizes of LSM in heterogeneous systems thus depend on both the adult egg-laying behaviour in response to LSM and the order that LSM is applied to the pools (Fig. 3c,d).

Not surprisingly, the control effect sizes of LSM would be substantially larger if LSM were targeted at the most productive pools (Fig. 3c,d), and it would be substantially less efficient if not. The most efficient solution – targeting the most productive pools in rank order of their productivity from most to least – results in sharp increase in control effect sizes for even small coverage levels, regardless of adult mosquito egg-laying behaviour. The analysis here suggests that the control effect sizes are greater than log-linear, such that it is possible to reduce transmission by a hundred-fold with moderate coverage through targeted, repeated application of modern (i.e., non-repellent) larvicides and other modes of LSM that create an egg sink effect among the most productive pools.

Like homogeneous systems, the outcome of LSM in heterogeneous systems is also dependent on egg-laying behaviour of mosquitoes in treated pools. The effects of efficient targeting are similar between both types of egg-laying responses, but control effect sizes are always higher, all else equal, when mosquitoes continue to lay eggs in treated pools. Like the homogeneous systems, the “egg-sink” effect in heterogeneous systems complements the removal effect to further reduce population densities (Fig. 3c,d). The magnitude of the egg sink effect varies, however, because the mismatch between productivity and the fraction of eggs laid means that the egg-sink effect is only approximately linear with respect to LSM coverage.

Control effect sizes are, on the other hand, highly variable as a function of coverage when LSM is applied to pools in a random sequence. For the same random sequence, control effect sizes are always higher when eggs are laid in treated pools (Fig. 3c,d). Even with perfectly inefficient targeting, control effect sizes in relation to coverage are nearly linear when eggs are laid in treated pools.

The outcomes of LSM were surprising, however, for some random sequences and for inefficient targeting in the case when adult mosquitoes avoid treated pools and redistribute eggs elsewhere (Fig. 3c,d). In the case of perfectly inefficient targeting (*i.e.*, when a subset of the least productive pools is treated), eggs are redistributed from less to more productive pools. The effect is counteracted by a reduction in total carrying capacity. The net effects change with coverage and with the particular distribution of pools (Fig. 3c,d). Similarly, for a random sequence of pools, productivity can increase as coverage increases whenever the effect of redistributing eggs to more productive habitats is greater than the loss of capacity. The behavioural responses of mosquitoes thus make it possible for LSM to increase overall mosquito density by forcing adult mosquitoes to redistribute eggs in more productive pools when egg-laying under natural conditions is highly inefficient.

## Discussion

We conclude that mosquito egg laying is an important factor for mosquito population dynamics and that it can have strong affects on the outcome of LSM. In our analyses, increasing coverage caused quadratic reductions in mosquito density if mosquitoes continued to lay eggs in treated pools. Therefore LSM has the potential to be a highly effective method of malaria control without extensive coverage. In particular, we predict that moderate coverage targeted at the most productive aquatic habitats can achieve reasonably large reductions in transmission. Some of these conclusions are inconsistent with statements from the recent WHO report, which was based on a Ross-Macdonald model perspective [5]. Moreover, recent evidence demonstrates that, in some circumstances, LSM has been effective in reducing clinical malaria outcomes [25]–[31], and for similar costs to those of IRS and long-lasting insecticidal nets (LLINs) [6], [32]–[34]. Given all these caveats, generalizations about LSM are likely to depend heavily on the local context for pathogen transmission and operational constraints.

Our results highlight the lack of attention paid to heterogeneity in mosquito population dynamics and in considering the outcome of LSM. Habitat heterogeneity and local density dependence change the way that dynamics in mosquito population are regulated and could play a role in creating or controlling transmission hotspots [35], [36]. The concept of carrying capacity in models with homogeneous habitat and logistic growth must be modified in light of the heterogeneous structure of aquatic habitats with local density dependence. Crowding could be highly uneven such that a few habitats would have very high larval densities while others would be scarcely populated. In general, adult mosquito population densities will differ from capacity unless there is fine-tuning in the relationship between the carrying capacities of aquatic habitats and the egg-laying patterns of adult mosquitoes. The rules governing mosquito population densities are more aptly described as a system in which crowding thins the aquatic mosquito populations.

When LSM is integrated into these models, egg-laying behaviour is identified, once again, as an important issue. The effects of LSM are approximately quadratic when mosquitoes continue to lay eggs in treated habitats, and these treated habitats function as egg sinks [23], [24]. This effect is quadratic because LSM has two distinct linear effects that could be created separately by first removing productive habitat, and second replacing that habitat with oviposition traps that absorb just as many eggs. Using larvicides that repel mosquitoes has only the first effect, while using larvicides that do not repel mosquitoes has both effects. The product of these two linear effects creates a non-linear response (i.e. quadratic) much like the one that Macdonald identified in his oft-used analysis [2], [4], [5]. This analysis also raises an operationally relevant question about the repellent effects of modern larvicides at concentrations ordinarily used for field application [37]–[46]. Targeting of these systems [47] can lead to disproportionate efficiencies in the effectiveness of LSM, though practical advice about how to identify productive larval habitats for targeting remains a critical need. In places where the aquatic habitats are in the same places year after year, is possible for control programs to learn and adapt to local systems [47]. Factors that may seem to present technical limitations for LSM – such as the need to target the most productive habitats – can be turned into a long-term operational advantage. It may be possible, for example, to accumulate knowledge about the local mosquito ecology and thereby improve the effectiveness of LSM over time.

The collective results of eleven decades of vector control have been mixed [1], [48]. Overall, our study, along with many others [49], emphasizes the important role of various kinds of heterogeneity in transmission dynamics and control. Heterogeneities can have a strong influence on the ability to measure transmission or predict the outcomes of control programs. A general point to be made is that outcomes probably depend on *R_0_*, but they also depend on specific aspects of human and vector behaviours in specific contexts. Interventions that have not been explicitly considered in the Ross-Macdonald model cannot be derived intuitively from the formula for *R_0_*. Instead, they must be explicitly modelled and integrated into the underlying theory. Though some simple points can be made about the likely effects of LSM, simple mathematical models can often be misleading unless they identify the appropriate sources of heterogeneity. Application of the theory to LSM in this and other modelling studies [7], [8], [12] is increasingly based on information about local mosquito ecology and its relation to transmission. Given these concerns, even though analysis of mathematical models can help to inform policy, but empirical evidence should perhaps play a stronger role in evaluation of policy and in making policy recommendations. The consideration of LSM, LLINs, IRS, spatial repellents, attractive sugar toxic baits, genetic strategies, oviposition traps, and other new vector control tools designed to reduce transmission of a pathogen by mosquitoes all lead to the realization that there will be some advantages and some disadvantages for each approach, and that intervention success will vary by the transmission context and the efficiency with which programs are implemented. In the context of increasingly widespread insecticide and drugs resistance, limitations in delivery and coverage, and national and international funding constraints, what is urgently needed is programmatic flexibility. Success in ever changing environments will depend on the capacity to select from a suite of options a package of interventions that is best suited for local to national and regional vector-borne diseases prevention goals [50], [51].

## Supporting Information

Table S1 (PDF)

Analysis S1 (DOCX)
