# Supplementary material for: Mosquito Population Regulation and Larval Source Management in Heterogeneous Environments
Source: PLoS One. 2013 Aug 7;8(8):e71247. doi: 10.1371/journal.pone.0071247 (PMC3737150; doi:10.1371/journal.pone.0071247)
Supplement: Table S1 — (PDF) [file pone.0071247.s001.pdf]

Name	Definition	Default Values
$\boldsymbol{M}$	# adult mosquitoes	Variable
$\boldsymbol{f}$	Blood feeding rate, per mosquito, per day	$0.3\ d^{-1}$
$\boldsymbol{g}$	Adult mortality rate, per mosquito, per day	$0.083\ d^{-1}$
$\boldsymbol{\nu}$	# female eggs laid, per female mosquito, per feeding cycle	25
$\boldsymbol{N}$	# aquatic habitats (pools)	30
$\boldsymbol{L_i}$	# larvae in the ith pool	Variable
$\boldsymbol{K_i}$	Carrying Capacity of the ith pool	$\sqrt[\sigma_i]{f\nu\alpha_i g^{-1} - (\alpha_i + \gamma_i)}/\psi_i$
$\boldsymbol{\Lambda_i}$	Productivity of the ith pool, # adults emerging per day	$\alpha_i K_i$
$\boldsymbol{\lambda}$	Net productivity, total # adults emerging per day	$\sum_i \alpha_i L_i$
$\boldsymbol{O_i}$	# Eggs per day laid in the ith pool	$f\nu p_i M$
$\boldsymbol{p_i}$	Proportion of eggs laid in the ith pool	$1/N$ or ||rbeta(1,10)||
$\boldsymbol{\alpha_i}$	Maturation rate of larvae in the ith pool	$0.1\ d^{-1}$
$\boldsymbol{\gamma_i + \psi_i L_i^{\sigma_i}}$	Death rate of larvae, per capita, in the ith pool	$\gamma_i = 0.1\ d^{-1}$or rbeta(1,9)<br>$\psi_i = 0.01\ M^{-1}$ or rbeta(1,19)<br>$\sigma_i = 1$
