# Supplementary material for: Mosquito Population Regulation and Larval Source Management in Heterogeneous Environments
Source: PLoS One. 2013 Aug 7;8(8):e71247. doi: 10.1371/journal.pone.0071247 (PMC3737150; doi:10.1371/journal.pone.0071247)
Supplement: Analysis S1 — (DOCX) [file pone.0071247.s002.docx]

**Supplementary Analysis**

The Jacobean of this system of equations is the following:

$$J=\left[ \begin{matrix} -\left( \alpha_{1}+ \gamma_{1}+\psi_{1}\sigma_{1}L_{1}^{\sigma_{1}-1} \right) & 0 & \ldots& 0 & fvp_{1} \\ 0 & -\left( \alpha_{2}+ \gamma_{2}+\psi_{2}\sigma_{2}L_{2}^{\sigma_{2}-1} \right) & \ldots& 0 & fvp_{2} \\ \vdots& \vdots& \ddots& 0 & \vdots\\ 0 & 0 & 0 & -\left( \alpha_{N}+ \gamma_{N}+\psi_{N}\sigma_{N}L_{N}^{\sigma_{N}-1} \right) & fvp_{N} \\ \alpha_{1} & \alpha_{2} & \ldots& \alpha_{N} & -g \end{matrix} \right].$$

The stability of this system is given by the values of the eigenvalues of $J$.

We focus on the number of adult females produced by a single adult female over her lifetime. A single female lays $fv/g$ eggs. When a pool is not crowded, a fraction $\alpha_{i}/ (\alpha_{i}+ \gamma_{i})$of the eggs laid in the i^th^ pool emerge to become adults, and a fraction of each females eggs $p_{i}$ is laid in that pool. From this, a threshold condition can be proposed based on the expected number of adult female mosquito offspring from a single adult female mosquito:

$$\frac{fv}{g}\sum_{i} \frac{p_{i}\alpha_{i}}{\alpha_{i}+\gamma_{i}}>1.$$

A sufficient condition is that at least one of these habitats is capable of sustaining itself from the eggs laid in that pool by an adult female emerging from that pool, regardless of where the other eggs are laid:

$$fvp_{i}\alpha_{i}>{g(\alpha}_{i}+\gamma_{i}).$$

The figures were all created in R. The values of the parameters and distributions used to create the figures are give in Table #S1.

**Table #S1:** Parameter names, terms, and default values used in the simulations. The distributions named are using the standard parameterizations of the distributions in R. The symbol $\left\| x \right\|$ means the values were normalized after being drawn from the distribution.
